# p38 Inhibition Decreases Tau Toxicity in Microglia and Improves Their Phagocytic Function

**DOI:** 10.1007/s12035-021-02715-0

**Published:** 2022-01-10

**Authors:** Juan R. Perea, Marta Bolós, Raquel Cuadros, Esther García, Vega García-Escudero, Félix Hernández, Róisín M. McManus, Michael T. Heneka, Jesús Avila

**Affiliations:** 1 Department of Molecular Neuropathology, Centro de Biología Molecular “Severo Ochoa” (UAM-CSIC), 28049 Madrid, Spain; 2 Center for Networked Biomedical Research On Neurodegenerative Diseases (CIBERNED), 28031 Madrid, Spain; 3 German Center for Neurodegenerative Diseases (DZNE), 53127 Bonn, Germany; 4 Department of Anatomy, Histology and Neurosciences, Faculty of Medicine, Universidad Autónoma de Madrid (UAM), 28029 Madrid, Spain; 5 Department of Molecular Biology, Faculty of Sciences, Universidad Autónoma de Madrid (UAM), 28049 Madrid, Spain

**Keywords:** Tau, p38, Microglia, Tauopathies, Alzheimer’s disease, Neuroinflammation

## Abstract

Alzheimer’s disease (AD) and other tauopathies are histopathologically characterized by tau aggregation, along with a chronic inflammatory response driven by microglia. Over the past few years, the role of microglia in AD has been studied mainly in relation to amyloid-β (Aβ) pathology. Consequently, there is a substantial knowledge gap concerning the molecular mechanisms involved in tau-mediated toxicity and neuroinflammation, thus hindering the development of therapeutic strategies. We previously demonstrated that extracellular soluble tau triggers p38 MAPK activation in microglia. Given the activation of this signaling pathway in AD and its involvement in neuroinflammation processes, here we evaluated the effect of p38 inhibition on primary microglia cultures subjected to tau treatment. Our data showed that the toxic effect driven by tau in microglia was diminished through p38 inhibition. Furthermore, p38 blockade enhanced microglia-mediated tau phagocytosis, as reflected by an increase in the number of lysosomes. In conclusion, these results contribute to our understanding of the functions of p38 in the central nervous system (CNS) beyond tau phosphorylation in neurons and provide further insights into the potential of p38 inhibition as a therapeutic strategy to halt neuroinflammation in tauopathies.

**Supplementary Information:**

The online version contains supplementary material available at 10.1007/s12035-021-02715-0.

## Introduction

Alzheimer’s disease (AD) is the most common form of dementia, affecting 47 million people worldwide. It is the main cause of dependence in older adults and the fifth cause of death. Although this disease has an incredibly high socio-economic burden, there are still no effective therapies [1, 2]. AD is characterized by the presence of senile plaques (comprised of amyloid-β peptide (Aβ)) [3–5] and neurofibrillary tangles (NFTs) (formed by the aggregation of hyperphosphorylated tau) [6–8] in the brain, which cause synaptic dysfunction, neuron loss and marked glial activation [9].

Tau is a microtubule-associated protein that regulates axonal transport and stabilizes and spatially organizes the microtubules of neurons [10, 11], among other functions [12]. Tau protein is subjected to numerous post-translational modifications, phosphorylation being the most recurrent [13]. This process is physiologically regulated throughout life. However, in some neurodegenerative diseases called tauopathies (such as AD), tau is hyperphosphorylated [14]. The state of phosphorylation plays a very important role in regulating the physiological function of tau, since it reduces the binding affinity of tau to microtubules and promotes its aggregation, thus compromising the integrity of neurons [15, 16]. On the other hand, several authors have reported that NFTs are not as toxic as previously believed, thereby suggesting that small aggregates and soluble tau species are the main drivers of neurodegeneration in tauopathies [17–25]. In this regard, various mechanisms can release tau into the extracellular space, where it interacts with other neurons [26–29] or glial cells such as astrocytes [30, 31] and microglia [32, 33].

Microglia are the resident macrophages of the brain [34, 35] and make up 5–12% of central nervous system (CNS) cells [36]. Alois Alzheimer was the first to recognize the involvement of glia in the disease that bears his name [37]. However, it was in the 1990s that microglia were shown to interact with Aβ and tau [38, 39]. ﻿For the last thirty years, the scientific community, supported by the amyloid hypothesis, has focused on studying the contribution of Aβ to neuroinflammation, while less attention has been paid to the association between microglia and tau pathology. In this context, Ising et al. recently showed that tau promotes NLRP3 inflammasome activation [40]. Moreover, previous work by our group demonstrated that tau induces a pro-inflammatory response in microglia through p38 MAPK activation [41].

Mitogen-activated protein kinase (MAPK) signal transduction pathways are ubiquitous and highly evolutionarily conserved mechanisms that coordinate and integrate responses to diverse stimuli (e.g., pathogen-associated molecular patterns (PAMPs), danger-associated molecular patterns (DAMPs), cytokines and environmental stresses) and are thus associated with various cellular processes [42]. The p38 MAPK subfamily, comprising four isoforms encoded by *MAPK14* (p38α), *MAPK11* (p38β), *MAPK12* (p38γ) and *MAPK13* (p38δ) [43], is one of the most important signaling pathways in inflammation [44]. p38 activation in AD [45] has been fundamentally attributed to its ability to phosphorylate tau in neurons [46–49], while its function in microglia has not been fully elucidated.

Here we show that extracellular tau exerts a toxic effect in microglia that is reversed by pharmacological inhibition of p38. Furthermore, p38 blockade promotes an increase in tau phagocytosis, although it diminishes tau-mediated microglial migration. These results support the notion that targeting p38 signaling might offer a potential therapeutic strategy through which to improve microglial function and prevent neuroinflammation in tauopathies.

## Materials and Methods

### Recombinant Tau Preparation

pRKT42, which encodes full-length tau protein of human origin (2N4R), was transformed into lipopolysaccharide (LPS)-free ClearColi (BL21(DE3)) cells (Lucigen, Cat#60810) following the manufacturer’s instructions. Single colonies were grown in LB medium with 100 µg/ml of ampicillin, and 0.4 mM IPTG was added when OD_600_ reached 0.6–0.8. Four hours later, bacteria were pelleted (20 min, 1,000 g, 4 °C) and sonicated in extraction buffer (0.1 M MES, 2 mM EGTA, 0.5 mM MgCl_2_, 0.5 M NaCl, 5 mM β-mercaptoethanol, and 1 mM PMSF). The sonicated product was then centrifuged (10 min, 23,700 g, 4 °C) and the supernatant was boiled for 10 min to prevent possible contamination of endogenous enzymatic activities. The boiled sample was incubated on ice for 5 min and subsequently centrifuged (30 min, 23,700 g, 4 °C). Tris was then added to the supernatant until pH = 11.

To induce tau precipitation, the sample was stirred for 1 h at 4 °C in a magnetic mixer and (NH_4_)_2_SO_4_ was added until reaching 50% saturation. Next, the sample was centrifuged (1 h, 23,700 g, 4 °C) and the pellet was resuspended in PBS. The resuspension was dialyzed in PBS with a Spectra/Por3 membrane (Repligen, Cat#132720) overnight at 4 °C in a magnetic mixer. The next day, the dialyzed product was incubated for 30 min at 37 °C while shaking with 10 µg/ml of RNase A (Roche, Cat#10109169001). To stop the enzymatic activity of the RNase and remove it, the sample was incubated for 1 min at 100 °C and centrifuged (1 min, 15,900 g, 4 °C).

To efficiently remove the remaining endotoxins, the supernatant was subjected to Triton X-114 phase separation. First, Triton X-114 was added at a concentration of 1% and the sample was incubated for 30 min at 4 °C in a rotating shaker with a vortex step every 5 min. The sample was then incubated for 10 min at 56 °C in a water bath and centrifuged (10 min, 20,000 g, 25 °C) to obtain the liquid phase. This procedure was repeated three times and the liquid phase of the third cycle was processed through a Pierce detergent removal spin column (Thermo Fisher, Cat#87779), following the manufacturer’s instructions. The eluted volume was passed through a polymyxin B-agarose (Sigma Aldrich, Cat#P1411) endotoxin removal column, and endotoxin levels were quantified by LAL assay (HyCult Biotech, RRID:AB_10130891).

For some experiments, purified tau was labeled with sulfoindocyanine Cy5 (GE Healthcare, Cat#PA25001), following the manufacturer’s recommendations. Briefly, 1 ml of recombinant tau protein (1 mg/ml), which comprises mainly monomers and dimers [50], was mixed with a sample of Cy5 dye for 1 h at room temperature. To remove the excess of free-dye, the mixture was dialyzed in PBS overnight at 4 °C and filtered through a Sephadex G-50 column. Finally, each eluted fraction was tested for the presence of tau by dot blot, and tau-containing fractions were combined.

### Primary Microglia Cultures

Mice were housed in 403 (W) × 165 (D) × 174 (H) mm cages (4–5 mice/cage) at the specific pathogen-free animal facility of the “Centro de Biología Molecular Severo Ochoa.” They had access to food and water ad libitum and were maintained on a 12/12 h light/dark cycle in a temperature-controlled environment. Animal housing and maintenance protocols followed the guidelines of the Council of Europe Convention ETS123, revised as indicated in Directive 86/609/EEC. Animal experiments were performed under protocols (P15/P16/P18/P22) approved by the Institutional Animal Care and Utilization Committee (PROEX 291/15) and following ARRIVE guidelines.

Microglia were cultured from newborn C57BL/6 J (Charles River, RRID:MGI:6151402) mice (P0-P3). Animals were decapitated, and brains were dissected in cold Ca^2+^/Mg^2+^-free HBSS and stripped of meninges. The tissue was then digested with 0.25% trypsin for 10 min at 37 °C. Trypsinization was stopped by the addition of the medium used for this cell culture: DMEM supplemented with 1% Glutamax (Gibco, Cat#35050–038), 10% FBS (Gibco, Cat#26140–079), 100 U/mL of penicillin and 0.1 mg/mL of streptomycin (Gibco, Cat#15240–062). Next, 0.2 mg/ml DNase (Roche, Cat#11284932001) was added to the digested tissue and centrifuged (10 min, 300 g, 4 °C). A single-cell suspension of the pellet was obtained by repeated pipetting. Subsequently, cells were passed through a 40-μm nylon filter and seeded into 75 cm^2^ flasks coated with 0.1 mg/ml poly-L-lysine (Sigma Aldrich, Cat#P9155) (2 brains/flask), which were maintained at 37 °C in humidified 5% CO_2_–95% air. The next day, the plating medium was discarded, the flasks were washed three times with PBS, and fresh medium supplemented with 10% L929 conditioned medium was added. Microglia were collected after 7–10 days by shake off and centrifugation (10 min, 300 g, room temperature).

### Live-Cell Imaging

One hundred thousand cells/well were seeded on an M24 plate. After 2 days, cells were treated with 1 µg/ml of LPS (from *E. coli* O55:B5, Sigma Aldrich, Cat#L2880), 0.5 µM tau, or PBS (control). Some of the experiments were subjected to a 30 min pre-treatment with 20 μM SB203580 (Sigma Aldrich, Cat#S8307), 10 μM cytochalasin D (Sigma Aldrich, Cat#C2618) or vehicle (DMSO). The plate was immediately placed in an Incucyte S3 system (Sartorius) and 9 images/well were taken every hour for 24–48 h with a 10 × objective. Automated image processing (Supplementary Fig. S1) and quantification were carried out by the integrated controller unit (Sartorius) using Incucyte S3 v.2017A software. The total sum of the Cy5 signal of all cells in each image ((RCU × µm^2^)/image) was calculated to analyze tau-Cy5 internalization by microglia. Cell death events were visualized by adding 250 nM Cytotox Red reagent (Sartorius, Cat#4632), following the manufacturer’s instructions. Confluence analysis was performed by calculating the percentage of area occupied by the cells.

### Western Blot

One hundred thousand cells/well were seeded on an M24 plate. After 2 days, cells were pre-treated for 30 min with 20 μM SB203580 (Sigma Aldrich, Cat#S8307), 10 μM cytochalasin D (Sigma Aldrich, Cat#C2618) or vehicle (DMSO), and 0.5 µM tau or PBS (control) was subsequently added for 30 min. Afterwards, cells were washed three times with PBS to remove excess tau. Finally, the samples were lysed using the RIPA buffer (50 mM Tris–HCl pH 7.4, 150 mM NaCl, 1% Triton X-100, 0.5% sodium deoxycholate, and 0.1% SDS) with a mixture of protease (cOmplete, Roche, Cat#11697498001) and phosphatase (0.1 mM okadaic acid and 5 mM orthovanadate) inhibitors. After a centrifugation step (5 min, 845 g, 4 °C), the protein concentration of the supernatant was determined using the BCA assay (Thermo Fisher, Cat#23225).

5 × Laemmli buffer (0.125 M Tris–HCl pH 6.8, 20% glycerol, 5% SDS, 0.004% bromophenol blue, and 2% β-mercaptoethanol) was added to the protein extracts, which were denatured at 100 °C for 5 min. Proteins were then separated by standard SDS-PAGE electrophoresis in 10–12% polyacrylamide gels. They were then transferred to polyvinylidene difluoride membranes (Millipore, Cat#IPVH00010) using a Mini-PROTEAN system (Bio-Rad) at a constant amperage of 150 mA for 50 min (90 min for Hsp27 and phospho-Hsp27 immunoblotting). Membranes were blocked with 5% BSA in 0.1% TBS-T for 2 h at room temperature and incubated overnight at 4 °C with primary antibodies. The following day, membranes were washed 3 times with 0.1% TBS-T and incubated with secondary antibodies for 2 h at room temperature. Finally, membranes were subjected to 3 washes with 0.1% TBS-T before being developed with ECL (PerkinElmer, Cat#NEL105001EA) and photographed on an ImageQuant LAS 4000 mini system (GE Healthcare). The signal intensity was quantified by densitometry using Fiji v.2.1.0/1.53c (RRID:SCR_002285). To correct for possible loading errors, the densitometry values obtained were normalized with loading controls.

### Lentiviral Stock Preparation

HEK 293 T cells (RRID:CVCL_1926. Authenticated by STR profiling, confirmed for mycoplasma negative, and not listed as commonly misidentified cell line by the International Cell Line Authentication Committee) were seeded at a passage number lower than 10 in 100-mm dishes and co-transfected at 85% confluency with 5 µg of the packaging plasmid pCMVR8.74 (RRID:Addgene_22036), 2 μg of the VSV-G envelope protein plasmid pMD2.G (RRID:Addgene_12259) and 5 µg of the corresponding lentivector plasmid pWPI (RRID:Addgene_12254) or pWPI-Tau (kindly provided by Prof. Kenneth S. Kosik. Encodes full-length tau (2N4R) and GFP reporter) using Lipofectamine Plus (Thermo Fisher, Cat#18324–012), following the manufacturer’s instructions. The next day, the transfection medium was replaced with 5 ml of fresh microglia medium. After 24 h, the medium was passed through a 0.45  μm low binding pore filter and working titers (1.06–1.73 × 10^6^ infective units/ml) were quantified by flow cytometry using a FACSCalibur 4CA (BD) and FlowJo v.10.4 (RRID:SCR_008520).

### Bv-2 Infection

The Bv-2 microglial cell line (RRID:CVCL_0182. Not listed as commonly misidentified cell line by the International Cell Line Authentication Committee) was grown in modified RPMI medium (10 mM HEPES, 4.5 g/l of glucose, and 1.5 g/l of sodium bicarbonate) supplemented with 2 mM L-glutamine, 1 mM sodium pyruvate, 50 μg/ml of gentamycin and 10% FBS (Gibco, Cat#26140–079). Two hundred thousand cells/well were seeded at a passage number lower than 10 on an M6 plate. Two days later, lentiviral vectors were used to transfect the cells at a MOI of 1 in the presence of 8 μg/ml of polybrene (Sigma Aldrich, Cat#TR-1003). The medium was replaced every day and Green fluorescent protein (GFP) reporter expression was visualized under a microscope after 72 h. The percentage of infection was quantified by flow cytometry using a FACSCalibur 4CA (BD) and FlowJo v.10.4 (RRID:SCR_008520). Cells were then treated with 0.5 µM tau or PBS (control) for 30 min. Afterwards, they were washed three times with PBS to remove excess tau. Finally, the samples were lysed using the RIPA buffer with a mixture of protease and phosphatases inhibitors (composition detailed above). After a centrifugation step (5 min, 845 g, 4 °C), the protein concentration of the supernatant was determined by the BCA assay (Thermo Fisher, Cat#23225).

### Tau Internalization Assay

Fifty thousand cells/well were seeded on an M96 black plate. After 2 days, cells were pre-treated for 30 min with 20 μM SB203580 (Sigma Aldrich, Cat#S8307) or vehicle (DMSO), and 0.5 µM tau-Cy5 or PBS-Cy5 (control) was subsequently added at different time points (from 0.5 to 6 h). The culture medium was then removed from the wells and cells were washed three times with PBS. Finally, Cy5 fluorescence was measured in an Infinite M200 PRO plate reader (Tecan, RRID:SCR_019033) at an excitation wavelength of 630 nm and emission wavelength of 680 nm.

### Lysosomal Mass Quantification

Three hundred thousand cells/well were seeded on an M24 plate. After 2 days, cells were pre-treated for 30 min with 20 μM SB203580 (Sigma Aldrich, Cat#S8307) or vehicle (DMSO), and 0.5 µM tau or PBS (control) was subsequently added for 6 h. 75 nM Lysotracker Red (Thermo Fisher, Cat#L7528) was then added for 45 min, and cells were washed twice with PBS. Trypsin was then used to lift cells from the plate and cells were again washed twice with PBS by centrifugation (5 min, 150 g, 4 °C). Finally, cells were resuspended in 200 μl of PBS and analyzed using a FACSCanto II flow cytometer (BD) and FlowJo v.10.4 (RRID:SCR_008520). 1 µg/ml DAPI (Merck, Cat#268298) was used to exclude dead cells from the analysis.

### Microglia Migration Assay

One hundred cells were seeded onto an 8-μm pore size insert (Merck, Cat#MCEP24H48). Inserts were placed into an M24 plate containing 300 µl of medium with 0.5 µM tau or PBS (control) and 20 μM SB203580 (Sigma Aldrich, Cat#S8307) or vehicle (DMSO). After 3 h of incubation, inserts were removed and the medium was transferred to 1.5 ml tubes, which were centrifuged (5 min, 150 g, 4 °C). Pellet was resuspended in 10 μl trypan blue and cell counting was carried out using a 10-well hemocytometer (Kova International, Cat#87144E).

### Immunofluorescence

Sixty thousand cells/well were seeded on an M24 plate with 12 mm coverslips. After 2 days, cells were pre-treated for 30 min with 20 μM SB203580 (Sigma Aldrich, Cat#S8307) or vehicle (DMSO), and 0.5 µM tau-Cy5 or PBS-Cy5 (control) was subsequently added for 6 h. Afterwards, cells were washed three times with PBS to remove excess tau-Cy5, fixed in 4% paraformaldehyde (Electron Microscopy Sciences, Cat#15710) for 15 min at room temperature and washed again three times with PBS. Cells were then permeabilized with PBS containing 0.2% Triton X-100 for 10 min at room temperature and washed three times with PBS. After 30 min of blocking with 1% BSA in PBS-T at room temperature, cells were washed three times with PBS and stained with phalloidin 488 (Thermo Fisher, Cat#A12379) at 1:300 dilution in PBS for 1 h at room temperature. Finally, cells were washed three times in PBS, nuclei were labeled with 1 µg/ml DAPI (Merck) for 10 min at room temperature and rinsed with PBS.

Images were obtained using the SpinSR10 confocal system coupled to an IX83 inverted microscope (Olympus) with a 40 × objective. Each image is an overlay of 37 stacks with a separation of 1 μm between them. The proportion of migrating cells (characterized by the presence of a large filopodium) was quantified manually using the Cell Counter tool of Fiji. The occupied area was measured by drawing the contour of each cell individually. Fifty cells were analyzed for each experimental condition of three independent experiments.

### Statistical Analysis

Data were evaluated using GraphPad Prism v.9.0.0 (RRID:SCR_002798) of at least three independent experiments. The presence of outliers was checked using the Grubbs test. Subsequently, Shapiro–Wilk and D’Agostino-Pearson tests were used to verify that the remaining values were adjusted to a normal distribution. The area under the curve (AUC) was calculated when analyzing a variable over time. For the comparison between two experimental groups, data were analyzed by Student’s *t* test (two-tailed). One and two-way analysis of variance (ANOVA) tests were applied to compare more than two experimental groups, and post hoc comparisons were performed using Tukey’s multiple comparison test. Details of the number of independent cell culture experiments (*n*), data representation (mean ± standard error of the mean (SEM)), the statistical test used, and significance levels are provided in the caption of each figure. This work is an exploratory study and it was not pre-registered. No randomization, sample calculation or blinding was performed.

## Results

### Tau Exerts a Toxic Effect on Microglia

**Fig. 1:**
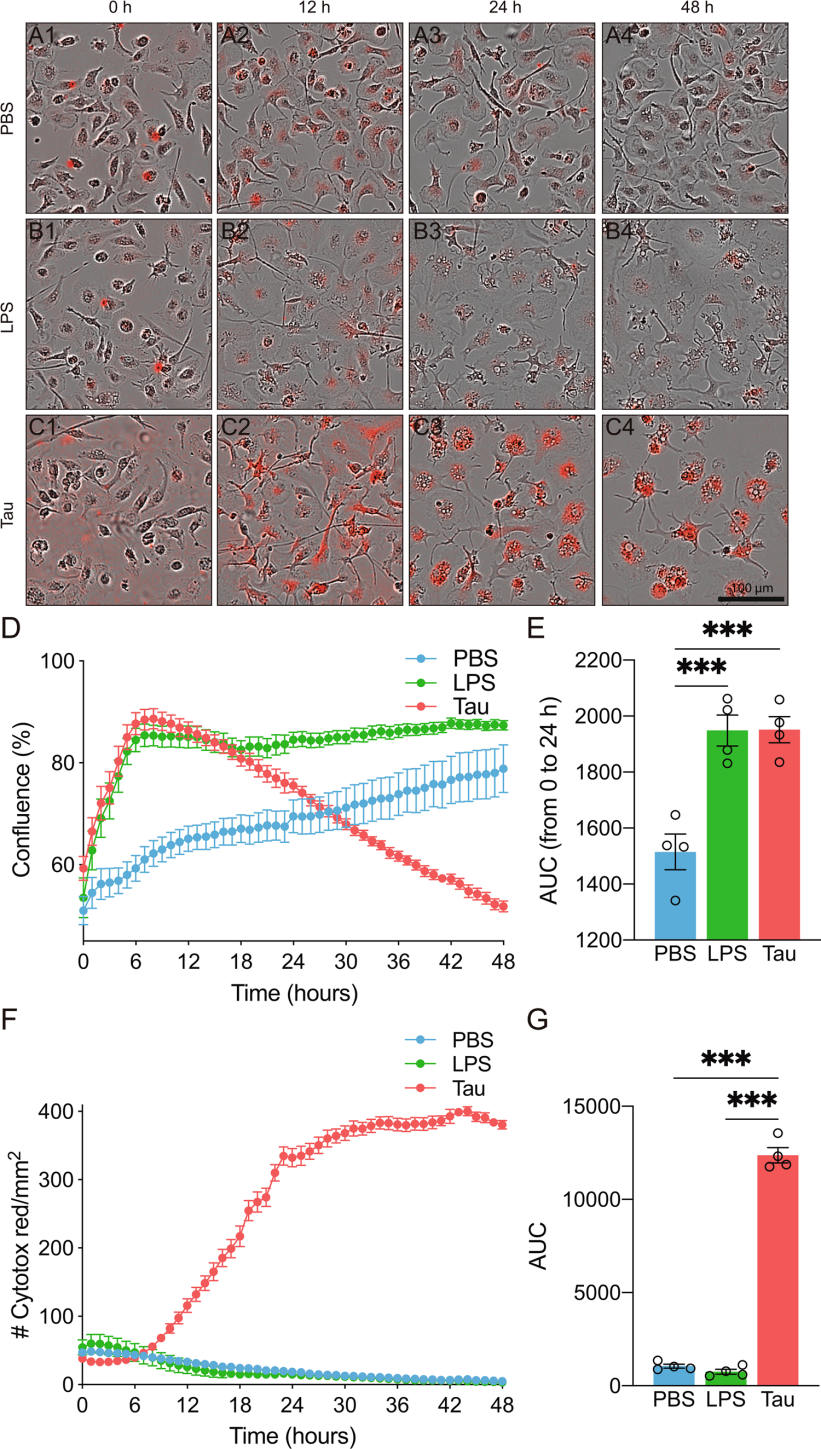
Tau exerts a toxic effect on microglia. Representative images of microglia treated with PBS (control) (**A**), LPS (**B**) or tau protein (**C**) for 48 h. Cytotox Red labeling indicates cytotoxicity. (**D**–**E**) Confluence analysis shows a dramatic increase in cell activation from 0 to 6 h with LPS and tau treatments. (**F**–**G**) Cytotoxicity analysis reveals that tau protein is toxic for microglia. *n* = 4. Graphs show mean ± SEM. ***p < 0.001 from one-way ANOVA. Scale bar, 100 µm. AUC, area under the curve; LPS, lipopolysaccharide; PBS, phosphate-buffered saline

We previously reported that extracellular tau is internalized by microglia [33, 51]. However, little is known about its consequences at the cellular level. Using live-cell imaging, we observed that microglia treated with tau of human origin acquired an amoeboid morphology and showed vacuoles, which is characteristic of an activated phenotype against harmful stimuli such as LPS (Fig. 1A–C). Confluence analysis also revealed a decrease in the area occupied by microglia in the presence of tau after 6 h of treatment compared with the LPS condition (Fig. 1D–E). This observation may indicate that tau compromises the survival of these cells. To address this issue, we used Cytotox Red, a highly sensitive cyanine nucleic acid dye that allows the evaluation of cell death. In this regard, we observed that the decrease in confluence in microglia subjected to tau treatment was accompanied by an increase in cell death (Fig. 1F–G and Supplementary video S1). This result confirms that tau exerts a toxic effect on microglia, as has been described in other cell types of the CNS such as neurons [52] (Supplementary Fig. S2). Given that cytotoxicity did not increase significantly between 24 and 48 h (Fig. 1F), the rest of the live-cell imaging experiments had a maximum duration of 24 h.

### Phagocytosis Inhibition Decreases Tau-Mediated Cytotoxicity in Microglia

**Fig. 2:**
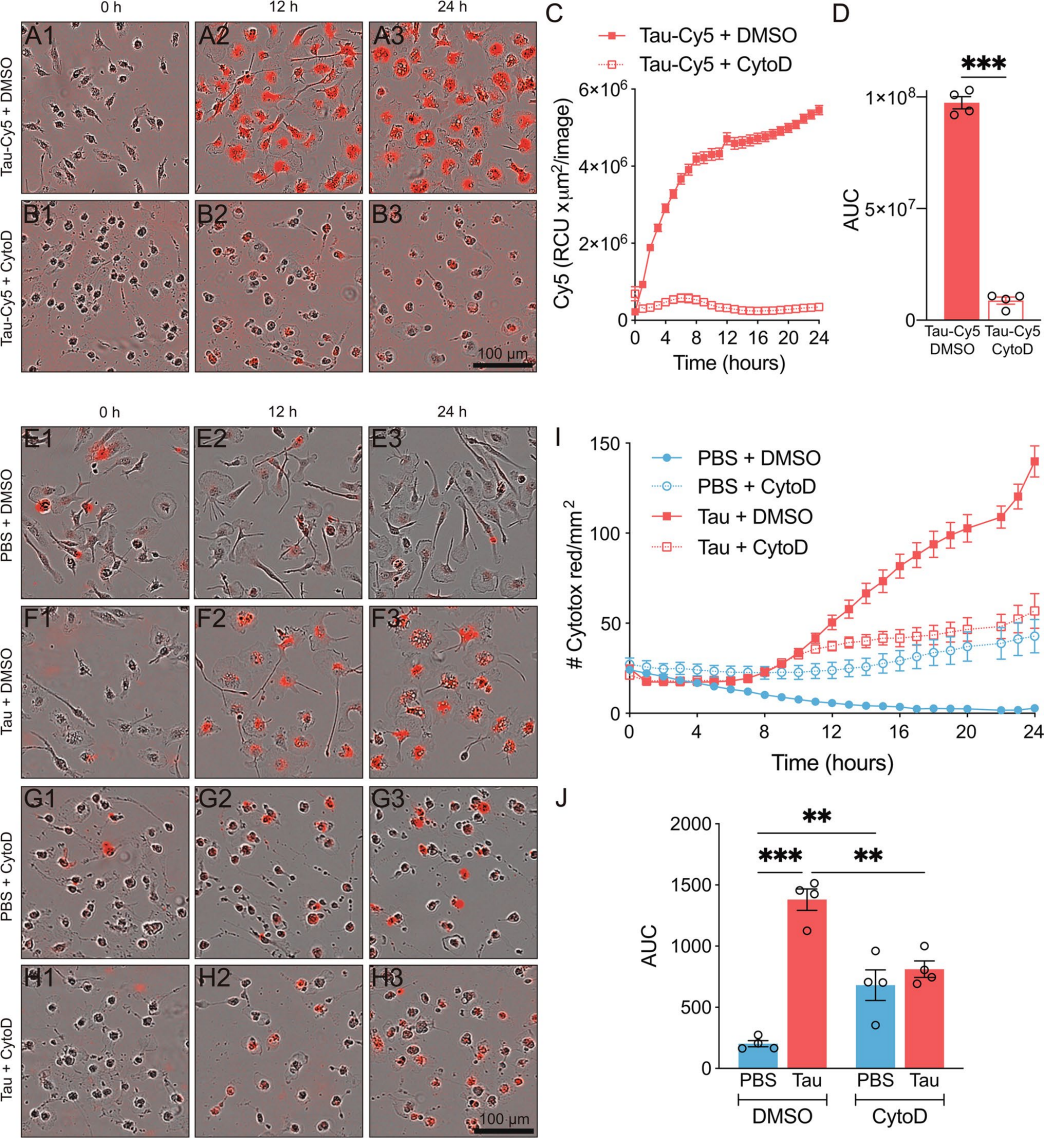
Phagocytosis inhibition decreases tau-mediated cytotoxicity in microglia. Representative images of microglia treated with tau-Cy5 (red) in the absence (**A**) or presence of cytochalasin D (CytoD) (**B**) for 24 h. (**C**–**D**) CytoD-mediated inhibition of phagocytosis prevents tau internalization. ***p < 0.001 from Student’s *t* test (two-tailed). Representative images of microglia treated for 24 h with PBS (control) (**E** and **G**) and tau protein (**F** and **H**) in the absence (**E**–**F**) or presence of CytoD (**G**–**H**). Cytotox Red labeling indicates cytotoxicity. (**I**–**J**) Cytotoxicity analysis shows that phagocytosis inhibition decreases tau-mediated cytotoxicity. **p < 0.01; ***p < 0.001 from one-way ANOVA. *n* = 4. Graphs show mean ± SEM. Scale bars, 100 µm. AUC, area under the curve; DMSO, dimethyl sulfoxide; PBS, phosphate-buffered saline; RCU, total red object integrated density

The toxic effect of tau protein could be due to its internalization by microglia. Phagocytosis is one of the essential functions of these cells since it intervenes in the correct development of the CNS and it is fundamental to eliminate pathogens and protein aggregates that accumulate with age [53]. In this context, tau can be released by neurons into the extracellular space and subsequently be internalized by neighboring cells [54]. To reinforce the notion that tau protein is phagocytosed by microglia, we subjected these cells to cytochalasin D (CytoD) treatment, a well-known phagocytosis inhibitor [55]. We next added tau labeled with Cy5 (tau-Cy5) to analyze the internalization of this protein, observing that phagocytosis inhibition prevented microglia-mediated tau uptake (Fig. 2A–D and Supplementary video S2). The diffuse signal observed in Fig. 2A and B corresponded to extracellular tau-Cy5 added to the cell culture prior to image acquisition. Also, to determine whether tau internalization gave rise to cell death, we analyzed cytotoxicity under phagocytosis inhibition. Although CytoD per se caused some toxicity, this drug significantly, but not totally, decreased tau-mediated cytotoxicity in microglia (Fig. 2E–J and Supplementary video S3).

### Tau Activates p38 Independently of Its Internalization by Microglia

**Fig. 3:**
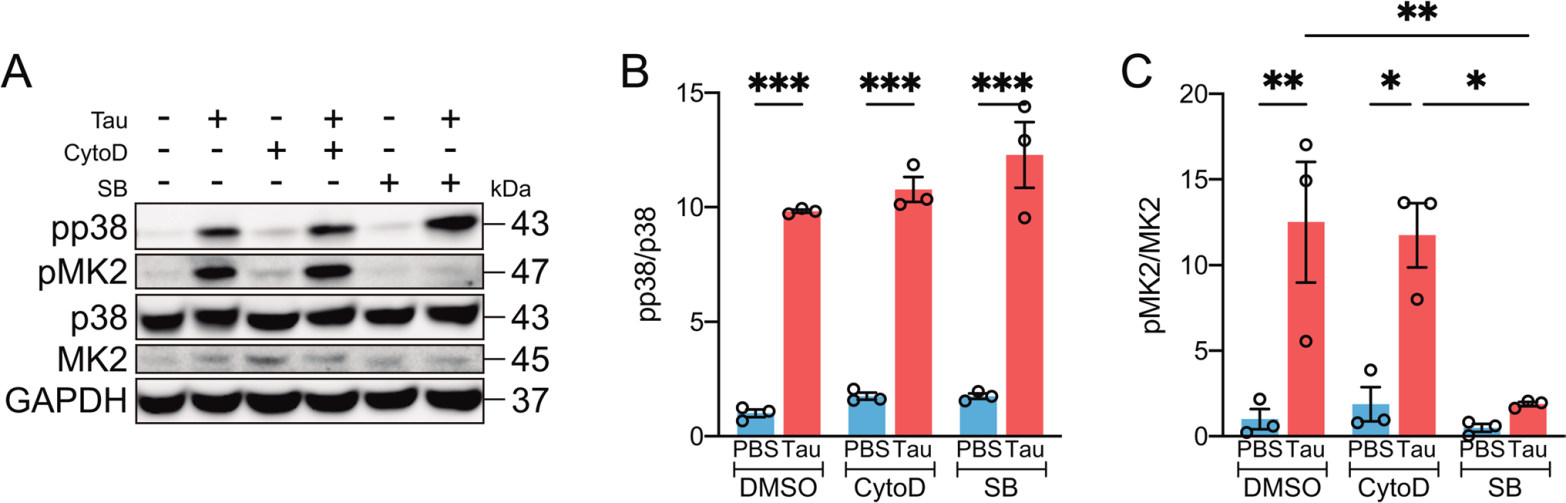
Tau activates p38 independently of its internalization by microglia. Western blot (**A**) and quantification of p38 (**B**) and MK2 (**C**) activity in microglia treated for 30 min with PBS (control) and tau in the absence or presence of cytochalasin D (CytoD) or SB203580 (SB). Note that only in the presence of SB is tau-mediated p38 activation suppressed, as shown by the measurement of MK2 activity. *n* = 4. Graphs show mean ± SEM. *p < 0.05; **p < 0.01; ***p < 0.001 from two-way ANOVA. DMSO, dimethyl sulfoxide; GAPDH, glyceraldehyde-3-phosphate dehydrogenase; MK2, MAPK-activated protein kinase 2; PBS, phosphate-buffered saline

After describing that phagocytosis blockade partially reduced the toxic effect of tau, we questioned whether the inflammatory response of these cells was also decreased under this condition. In this regard, p38 MAPK is one of the molecular triggers of inflammation [44], and previous work by our group demonstrated that tau induces p38 activation in microglia [41]. Taking this into consideration, we sought to determine whether the inhibition of tau phagocytosis reduced p38 activation. To this end, microglia were subjected to a 30 min pre-treatment with CytoD or SB203580 (SB), the latter a widely used p38 inhibitor. The cells were then incubated in the presence of tau for a further 30 min. Western blot analysis (Fig. 3A) showed that both p38 (Fig. 3B) and its downstream target MAPK-activated protein kinase 2 (MK2) (Fig. 3C) were activated despite blocking phagocytosis. This result indicates that tau promotes the activation of microglia without the need for its internalization.

### p38 Is Specifically Activated in the Presence of Extracellular Tau

**Fig. 4:**
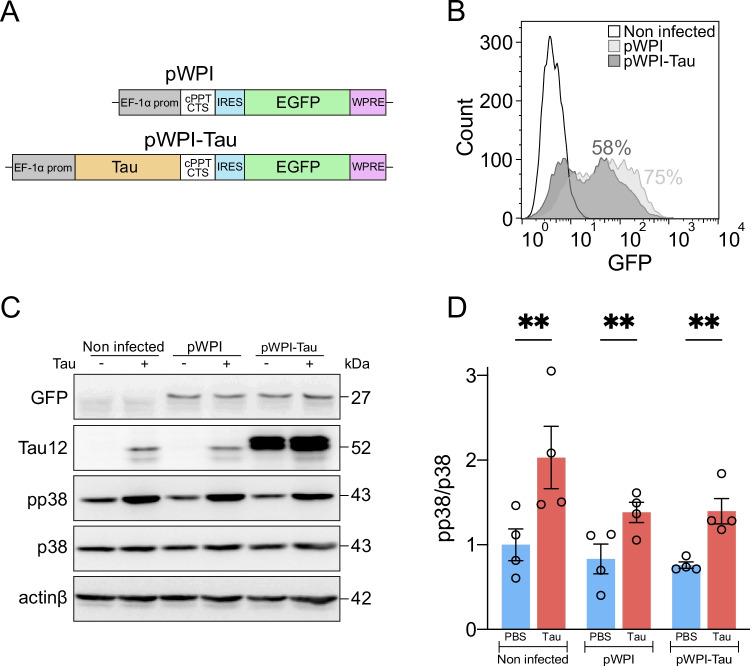
p38 is specifically activated in the presence of extracellular tau. (**A**) pWPI (control) and pWPI-Tau lentiviral constructs used to induce tau protein expression in Bv-2 microglial cells. (**B**) GFP reporter was used to quantify the percentage of infection by flow cytometry. Western blot (**C**) and quantification of p38 activity (**D**) in non-infected, pWPI-infected (control) and pWPI-Tau-infected Bv-2 cells treated for 30 min with PBS (control) and tau protein. Note that lentiviral infection and endogenous tau expression do not trigger p38 activation. However, the addition of tau to the extracellular medium does achieve this activation. *n* = 4. Graph shows mean ± SEM. **p < 0.01 from two-way ANOVA. cPPT/CTS, central polypurine tract/central termination sequence; EF-1α, elongation factor-1α; EGFP, enhanced green fluorescent protein; IRES, internal ribosome entry site; PBS, phosphate-buffered saline; WPRE, woodchuck hepatitis virus posttranscriptional regulatory element

We also wanted to demonstrate whether endogenous tau expression in microglia (that do not tau express per se [56, 57]) activates p38 in these cells. For this purpose, we made use of a lentiviral vector (pWPI-Tau) (Fig. 4A) that allows the efficient expression of tau in Bv-2 mouse microglia, as reported by the GFP reporter gene by flow cytometry (Fig. 4B) and western blot (Fig. 4C). Infected cells and their corresponding controls were treated with tau for 30 min, and p38 activity was quantified by western blot (Fig. 4C), showing that this kinase was activated exclusively in the presence of exogenous tau (Fig. 4D).

### p38 Inhibition Decreases Tau-Mediated Cytotoxicity in Microglia

**Fig. 5:**
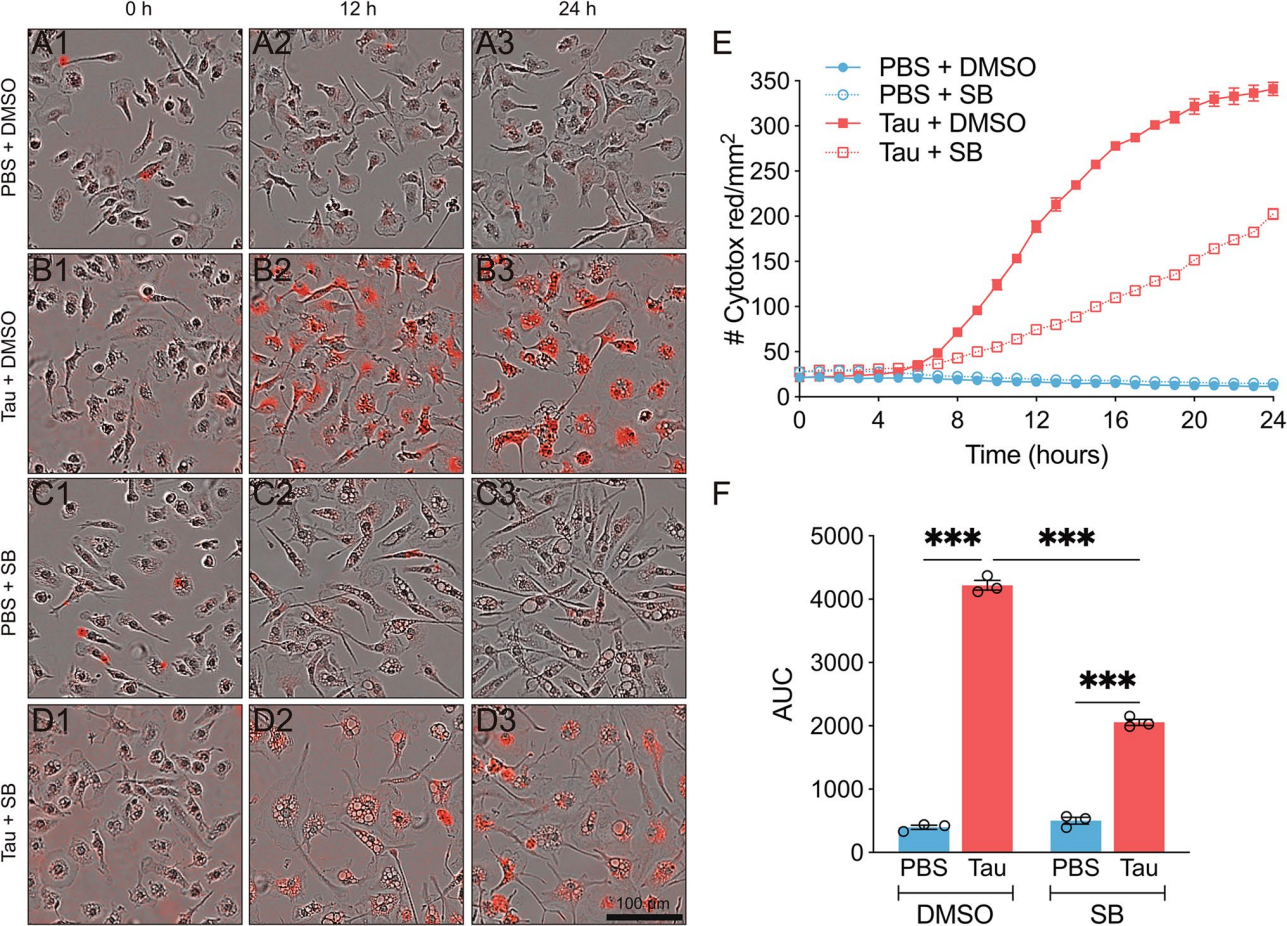
p38 inhibition decreases tau-mediated cytotoxicity in microglia. Representative images of microglia treated for 24 h with PBS (control) (**A** and **C**) or tau (**B** and **D**) in the absence (**A**–**B**) or presence of SB203580 (SB) (**C**–**D**). Cytotox Red labeling indicates cytotoxicity. (**E**–**F**) Cytotoxicity analysis shows that p38 inhibition decreases tau-mediated cytotoxicity. *n* = 3. Graphs show mean ± SEM. ***p < 0.001 from two-way ANOVA. Scale bar, 100 µm. AUC, area under the curve; DMSO, dimethyl sulfoxide; PBS, phosphate-buffered saline

Thus far, we have reported that the inhibition of tau phagocytosis decreased cytotoxicity in microglia (Fig. 2). However, this approach did not prevent p38 activation in these cells (Fig. 3). At this point, we sought to explore the possible beneficial effect of p38 inhibition on tau-mediated cytotoxicity. To this end, microglia were treated with SB for 30 min and tau was subsequently added for 24 h. Cytotox Red analysis revealed that SB per se had no toxic effect on these cells (Fig. 5A, C, E–F). Moreover, we confirmed that tau promoted an increase in cell death (Figs. 5A–B, E–F), as we had previously observed (Fig. 1 and 2). However, tau-mediated cytotoxicity was considerably reduced when SB was present (Fig. 5B, D–F and Supplementary video S4). The analysis of cell death by flow cytometry confirmed these results (Supplementary Fig. S3), thereby indicating that p38 inhibition alleviates cytotoxicity in microglia treated with tau.

### p38 Inhibition Enhances Tau Phagocytosis by Microglia

**Fig. 6:**
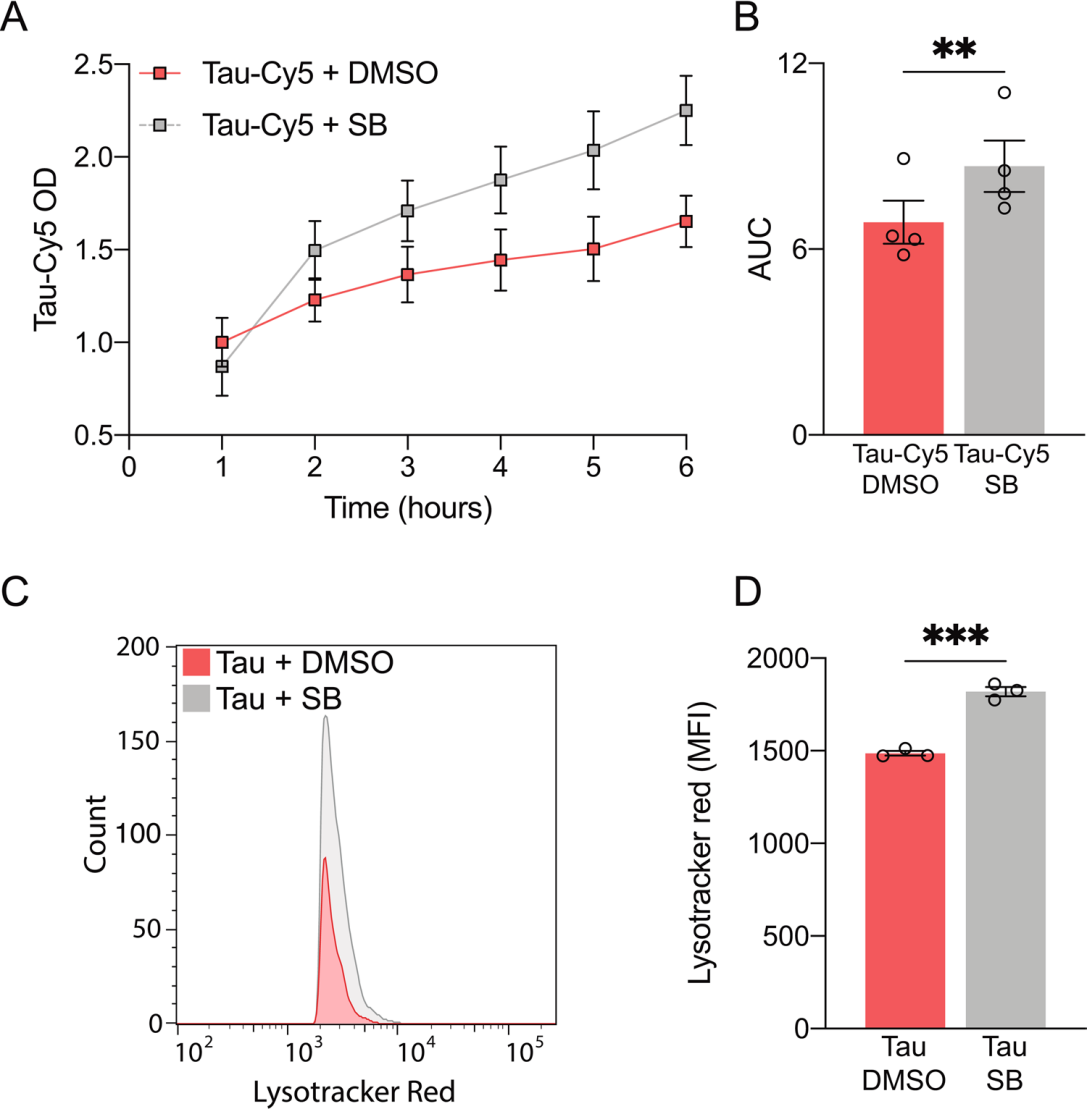
p38 inhibition enhances tau phagocytosis by microglia through an increase in lysosomal mass. (**A**–**B**) Time course of tau internalization by microglial cells in the absence or presence of SB203580 (SB). (**C**–**D**) Lysotracker red quantification reveals an increase in lysosomal mass when p38 is inhibited in microglial cells. *n* = 3–4. Graphs show mean ± SEM. **p < 0.01; ***p < 0.001 from Student’s *t* test (two-tailed). AUC, area under the curve; DMSO, dimethyl sulfoxide; MFI, mean fluorescence intensity; OD, optic density

Given that tau accumulates in the extracellular space in certain neurodegenerative diseases [58] and that the phagocytic capacity of microglia decreases with age [59], the discovery of potential targets that enhance phagocytosis is crucial to ensure the maintenance of CNS homeostasis. In this context, we analyzed the effect of p38 inhibition on tau phagocytosis by microglia. Cells were subjected to a 30 min pre-treatment with SB, and tau-Cy5 was subsequently added at different time points. A period between 1 and 6 h was chosen since there was not a drastic increase in cell death during this time interval (Fig. 1F). We observed that p38 inhibition increased tau phagocytosis in microglia (Fig. 6A–B). These data were further confirmed by flow cytometry (Supplementary Fig. S4).

In view of these results, we next sought to identify the mechanism responsible for this increase in tau internalization. Stancu et al. reported that tau is taken up by microglia and sorted to lysosomes [60]. Keeping this in mind, we studied whether there was an increase in the number of lysosomes. Using the Lysotracker Red probe, we detected an increase in lysosomal mass when cells were treated with SB (Fig. 6C–D). This observation thus suggests that p38 inhibition enhances tau phagocytosis through an increase in lysosome number.

### p38 Inhibition Partially Diminishes Tau-Mediated Microglial Migration

**Fig. 7:**
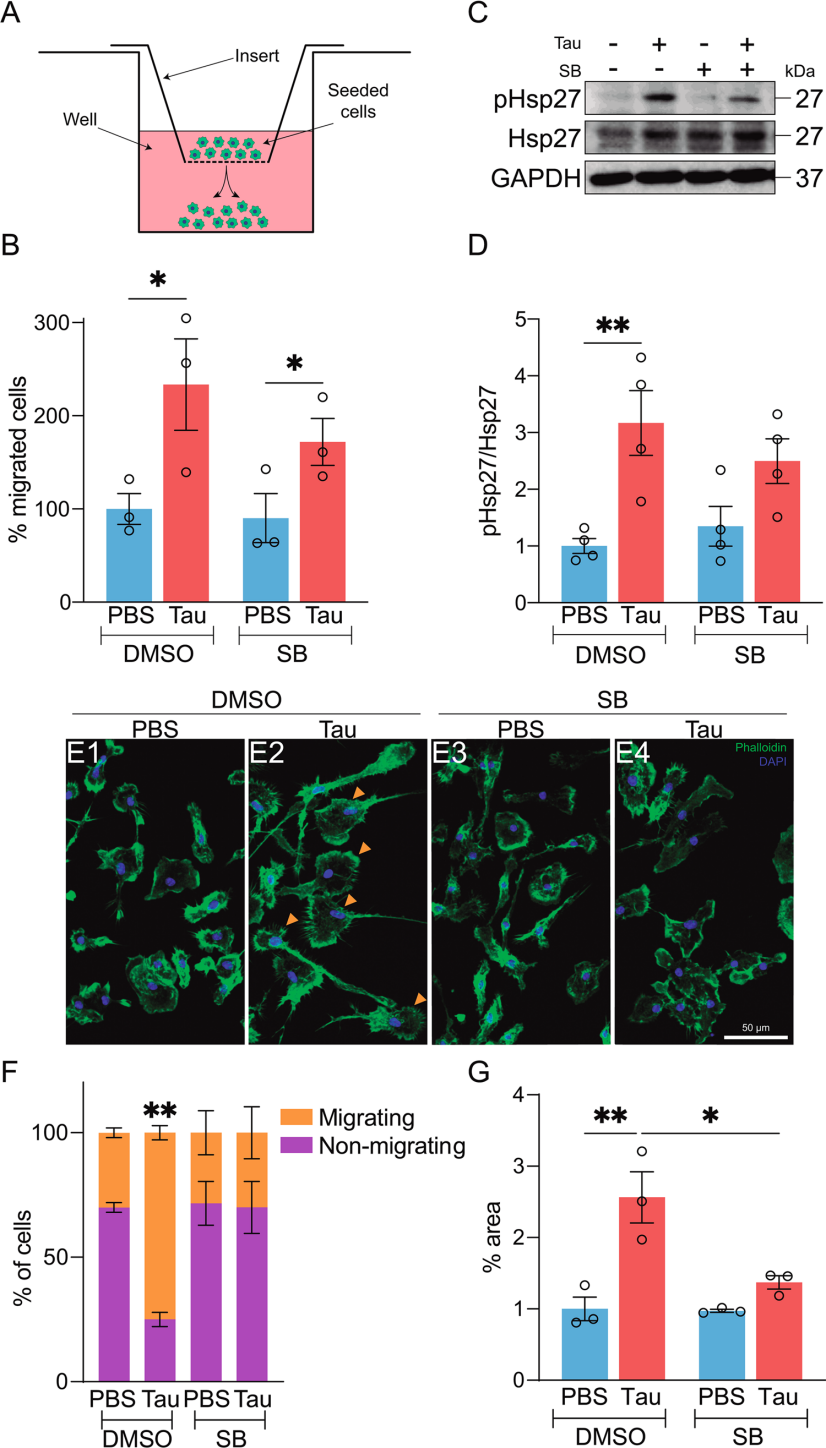
p38 inhibition diminishes tau-mediated microglial migration. (**A**) Representative diagram of the device used for the migration assay. The cells are seeded on the insert through which they can gradually migrate towards the base of the well. (**B**) Migration analysis shows that tau promotes microglial migration. However, when tau and SB203580 (SB) are added simultaneously, migration is reduced. Western blot (**C**) and quantification of Hsp27 activity (**D**) in microglia treated for 30 min with PBS (control) and tau in the absence or presence of SB. (**E**) Representative images of microglia stained with phalloidin. Orange arrows indicate migrating cells, which are characterized by the presence of a large filopodium. Quantification of the proportion of migrating vs. non-migrating cells (**F**) and the percentage of area occupied (**G**). *n* = 3–4. Graphs show mean ± SEM. *p < 0.05; **p < 0.01 from two-way ANOVA. Scale bar, 50 µm. DMSO, dimethyl sulfoxide; GAPDH, glyceraldehyde-3-phosphate dehydrogenase; Hsp27, heat shock protein 27; PBS, phosphate-buffered saline

Microglia constantly survey their environment thanks to their high motility, thus allowing them to rapidly detect changes in the brain parenchyma [61, 62]. During AD, NFTs (formed by the aggregation of hyperphosphorylated tau) act as an inflammatory focus, causing the surrounding microglia to approach the damaged area and exert a protective function [38]. However, studies analyzing the migration of microglia in the presence of tau are lacking. To address this gap, we performed an in vitro cell migration assay, which consisted of seeding the cells on an insert placed in a well with that its corresponding treatment (Fig. 7A). After 3 h of incubation under different experimental conditions, a significant increase in microglial migration in the presence of tau was observed. Nonetheless, simultaneous addition of SB and tau reduced the migration of these cells (Fig. 7B). To provide a possible explanation for this phenomenon, we analyzed the activity of heat shock protein 27 (Hsp27), a downstream target of p38 that regulates actin dynamics and, therefore, cell motility [63]. In this regard, western blot analysis showed an increase in Hsp27 activity when microglia were subjected to tau treatment. However, the addition of SB slightly reduced this activation (Fig. 7C–D). Additionally, a phalloidin staining was performed in order to analyze the dynamics of the actin cytoskeleton (Fig. 7E). When microglia were subjected to tau treatment, an increase in the proportion of migrating cells was observed (Fig. 7F). These cells are characterized by the presence of a large filopodium (orange arrows in Fig. 7E2) and their cytoplasmatic protrusions called filopodia. Consequently, microglia also increased their occupied area (Fig. 7G). However, as it was observed with the in vitro assay, the inhibition of p38 considerably reduced tau-mediated microglial migration.

## Discussion

Tau pathology is a hallmark of several neurodegenerative diseases whose incidence has grown rapidly in recent years due to the aging of the world population. Current research has focused on the role of tau in health and disease, as well as on strategies to mitigate tau accumulation, prevent its aggregation and promote its clearance [64]. However, there is a substantial knowledge gap concerning the molecular pathways involved in tau-mediated toxicity and neuroinflammation, thus hindering the identification of potential therapeutic targets for tauopathies such as AD. During the progression of this neurodegenerative disorder, neurons release various components into the extracellular space, including tau, usually as a result of neuronal death. However, these cells also present different mechanisms for the physiological release of tau, thus contributing to its propagation [54]. In this context, extracellular tau has a toxic effect on neuronal cells [52]. However, the repercussions on other cell types of the CNS, such as microglia, remain unexplored. To address this knowledge gap, we made use of primary microglia cultures in combination with live-cell imaging techniques and several in vitro assays to study tau toxicity in this cell population. While previous research shed light on the mechanism of tau internalization by microglia [32, 33, 51], this is the first study evidencing the toxic effect of extracellular tau on these cells. In this regard, inhibition of phagocytic function decreased tau-mediated cytotoxicity in microglia. However, the blockade of tau internalization did not prevent microglia from triggering an inflammatory response, as revealed by p38 activation.

The p38 MAPK signaling pathway is involved in a plethora of functions [43]. Although this kinase is expressed mainly in glia [56, 57], its role in the CNS has been associated with tau phosphorylation in neurons [65–67]. Nonetheless, recent research conducted by our group revealed that tau triggers an inflammatory response in microglia through p38 activation [41]. Also, that study showed that p38 inhibition reduces the levels of pro-inflammatory cytokines in microglia treated with tau. However, the effect of p38 blockade on tau-mediated cytotoxicity in these cells remained unknown. To address this question, here we demonstrate that p38 inhibition through SB203580 (SB) treatment decreases tau-mediated cytotoxicity in microglia. This finding is in agreement with previous observations by Xia et al., who determined that p38 signaling is critical for the induction of cell death processes [68].

Since p38 inhibition markedly reduced the mortality of microglia, we next explored whether SB treatment had additional effects on certain functions of these cells, such as phagocytosis or migration. Regarding the former, the CNS requires an efficient system that eliminates potentially harmful components present in the extracellular space [53]. In this context, microglia can internalize tau in vitro and in vivo [33], and here we show that p38 inhibition increased tau phagocytosis by these cells. This finding is in line with those of other studies which indicate that enhanced microglial phagocytosis may offer an effective therapeutic strategy during aging and in several neurodegenerative diseases [32, 69, 70]. Conversely, Reed-Geaghan et al. reported that p38 inhibition reduces Aβ phagocytosis [71]. This opposing effect could be attributed to distinct Aβ- and tau-associated microglial profiles in AD patients, which would support the notion that these cells have distinct phenotypes depending on the histopathological hallmark present [72].

In addition, this improvement in phagocytic function must be accompanied by effective molecular machinery capable of degrading toxic proteins. Consistent with this idea, recent studies reported that extracellular tau is internalized by microglia and sorted to lysosomes, which play an essential role in tau clearance [60, 73]. Taking this into consideration, we observed that p38 inhibition promoted an increase in lysosomal mass, thereby indicating that SB treatment enhanced tau phagocytosis through an increment in the number of lysosomes. In line with these findings, several studies support the notion that p38 signaling plays a key function in the endocytic pathway [74–76]. However, little is known about the role of p38 and its specific inhibitors in lysosomal biogenesis [77]. In this sense, more research is needed to elucidate the precise molecular mechanisms that induce an increase in tau internalization. Such information would then help to identify potential therapeutic targets for tauopathies [78].

Regarding microglial migration, both senile plaques and NFTs act as inflammatory foci, thus causing surrounding microglia to approach them and exert their protective function [38, 79]. While several authors have analyzed microglial migration in the context of Aβ [80–82], studies that describe this phenomenon in the presence of tau are lacking. In this regard, we report that tau induced microglia migration in vitro. This observation, together with the ability of these cells to degrade tau [83], supports the neuroprotective role of microglia in tauopathies. However, the migratory capacity of microglia decreases with age [84, 85], and it is crucial to find strategies to strengthen this feature. Accordingly, we examined whether p38 inhibition could fulfill this function. Contrary to what was expected, we found that SB treatment decreased microglial migration. This observation could be explained by the fact that p38 activation promotes actin polymerization and, consequently, cell motility [63]. Based on this evidence, we analyzed the activity of Hsp27, an F-actin capping protein whose phosphorylation gives rise to actin polymerization [86]. We found that the enhanced microglial migration caused by the presence of extracellular tau was accompanied by an increase in the Hsp27 phosphorylation, while p38 inhibition had the opposite effect. These observations support the notion that p38 activation during inflammation serves as a homeostatic function to regulate actin dynamics, which would otherwise be destabilized during disease processes [63]. However, although p38 inhibition reduces microglial migration, this pharmacological treatment also has other beneficial effects capable of counteracting tau toxicity, namely, attenuation of inflammation, reduction in cell death and improvement of phagocytic function.

Another significant finding of this study is that endogenous tau expression did not lead to p38 activation, meaning that this signaling pathway is activated only when extracellular tau interacts with distinct membrane receptors. In this regard, there is previous evidence about the interaction of tau with the plasma membrane [87] and recent studies have characterized the binding of tau with several receptors in neurons (LRP1, M1 and M3) [27, 88] and astrocytes (αV/β1) [89]. Conversely, the interaction of tau with microglial receptors is poorly understood and it has only been described that tau binds to a specific microglia receptor named C-X3-C chemokine receptor 1 (CX3CR1) [51]. Zhuang et al. demonstrated that the natural ligand (C-X3-C chemokine ligand 1. CX3CL1) of this receptor triggers p38 activation in microglia [90]. Surprisingly, we found that extracellular tau also activated this kinase in *Cx3cr1*^*−/−*^ microglia (Supplementary Fig. S5). Taken together, these results suggest that tau interacts with other receptors involved in the p38 signaling cascade. In this sense, it is plausible that tau could be interacting with receptors of the innate immune system such as Toll-like or complement receptors, whose association with Aβ has already been described [91, 92]. However, studies based on the analysis of tau interactome have not yet identified any potential candidates [93–95]. Therefore, more research is needed to provide additional clues to understand the molecular mechanism underlying tau toxicity in microglia.

Current research efforts are focused on defining the dynamics of p38 activation [96, 97] with the aim of developing more efficient compounds. Accordingly, several p38 inhibitors have been tested in animal models of Aβ accumulation [98–100], while less attention has been paid to tau pathology [101], in which we have recently shown that p38 activation occurs predominantly in microglia (in preparation). Regarding human clinical trials, neflamapimod (formerly known as VX-745) is the only p38 inhibitor that has completed phase 2 studies in patients with mild AD. The results of those trials indicated that a 24-week treatment did not improve episodic memory, although it caused a modest decrease in cerebrospinal fluid biomarkers associated with synaptic dysfunction. These findings thus suggest that a longer treatment at a higher dose would be needed to assess the effects on AD progression [102]. On the basis of our findings, future studies in humans require a distinction between Aβ, tau pathology, or their combination, to provide a comprehensive view of the effects of p38 inhibition in these patients.

In conclusion, our results make a significant contribution to enhancing our understanding of the dysregulated molecular mechanisms underlying tau toxicity in microglia. Moreover, our data reveal that p38 inhibition reduces the levels of pro-inflammatory cytokines, decreases cell death, and stimulates the phagocytic function of microglia, thus having a great impact on the resolution of neuroinflammation and extracellular tau clearance during tau pathology. Given these effects, p38 inhibition emerges as a potential therapeutic strategy for tauopathies.

## Supplementary Information

Below is the link to the electronic supplementary material.

Supplementary file1 (PDF 39777 KB)

Supplementary file2 (AVI 44077 KB)

Supplementary file3 (AVI 12805 KB)

Supplementary file4 (AVI 24066 KB)

Supplementary file5 (AVI 33170 KB)

## Data Availability

The data that support the findings of this study are available from the corresponding author upon reasonable request.

## References

[1] Wimo A, Guerchet M, Ali G-C (2017). The worldwide costs of dementia 2015 and comparisons with 2010. Alzheimers Dement.

[2] GBD (2016). Dementia Collaborators (2019) Global, regional, and national burden of Alzheimer’s disease and other dementias, 1990–2016: a systematic analysis for the Global Burden of Disease Study 2016. Lancet Neurol.

[3] Masters CL, Simms G, Weinman NA (1985). Amyloid plaque core protein in Alzheimer disease and Down syndrome. Proc Natl Acad Sci USA.

[4] Jarrett JT, Berger EP, Lansbury PT (1993). The C-terminus of the beta protein is critical in amyloidogenesis. Ann N Y Acad Sci.

[5] Maggio JE, Stimson ER, Ghilardi JR (1992). Reversible in vitro growth of Alzheimer disease beta-amyloid plaques by deposition of labeled amyloid peptide. Proc Natl Acad Sci USA.

[6] Kidd M (1963). Paired helical filaments in electron microscopy of Alzheimer’s disease. Nature.

[7] Crowther RA, Wischik CM (1985). Image reconstruction of the Alzheimer paired helical filament. EMBO J.

[8] von Bergen M, Friedhoff P, Biernat J (2000). Assembly of tau protein into Alzheimer paired helical filaments depends on a local sequence motif ((306)VQIVYK(311)) forming beta structure. Proc Natl Acad Sci USA.

[9] Serrano-Pozo A, Frosch MP, Masliah E, Hyman BT (2011). Neuropathological alterations in Alzheimer disease. Cold Spring Harb Perspect Med.

[10] Weingarten MD, Lockwood AH, Hwo SY, Kirschner MW (1975). A protein factor essential for microtubule assembly. Proc Natl Acad Sci USA.

[11] Dixit R, Ross JL, Goldman YE, Holzbaur ELF (2008). Differential regulation of dynein and kinesin motor proteins by tau. Science.

[12] Perea JR, Bolós M, Avila J (2020). Microglia in Alzheimer’s disease in the context of tau pathology. Biomolecules.

[13] Tapia-Rojas C, Cabezas-Opazo F, Deaton CA (2019). It’s all about tau. Prog Neurobiol.

[14] Rösler TW, Tayaranian Marvian A, Brendel M (2019). Four-repeat tauopathies. Prog Neurobiol.

[15] Grundke-Iqbal I, Iqbal K, Tung YC (1986). Abnormal phosphorylation of the microtubule-associated protein tau (tau) in Alzheimer cytoskeletal pathology. Proc Natl Acad Sci U S A.

[16] Biernat J, Gustke N, Drewes G (1993). Phosphorylation of Ser262 strongly reduces binding of tau to microtubules: distinction between PHF-like immunoreactivity and microtubule binding. Neuron.

[17] Bondareff W, Mountjoy CQ, Roth M, Hauser DL (1989). Neurofibrillary degeneration and neuronal loss in Alzheimer’s disease. Neurobiol Aging.

[18] Morsch R, Simon W, Coleman PD (1999). Neurons may live for decades with neurofibrillary tangles. J Neuropathol Exp Neurol.

[19] Kuchibhotla KV, Wegmann S, Kopeikina KJ (2014). Neurofibrillary tangle-bearing neurons are functionally integrated in cortical circuits in vivo. Proc Natl Acad Sci USA.

[20] Andorfer C, Acker CM, Kress Y (2005). Cell-cycle reentry and cell death in transgenic mice expressing nonmutant human tau isoforms. J Neurosci.

[21] Santacruz K, Lewis J, Spires T (2005). Tau suppression in a neurodegenerative mouse model improves memory function. Science.

[22] Sydow A, Van der Jeugd A, Zheng F (2011). Tau-induced defects in synaptic plasticity, learning, and memory are reversible in transgenic mice after switching off the toxic Tau mutant. J Neurosci.

[23] Van der Jeugd A, Hochgräfe K, Ahmed T (2012). Cognitive defects are reversible in inducible mice expressing pro-aggregant full-length human Tau. Acta Neuropathol.

[24] Cowan CM, Mudher A (2013). Are tau aggregates toxic or protective in tauopathies?. Front Neurol.

[25] Bengoa-Vergniory N, Velentza-Almpani E, Silva AM (2021). Tau-proximity ligation assay reveals extensive previously undetected pathology prior to neurofibrillary tangles in preclinical Alzheimer’s disease. Acta Neuropathol Commun.

[26] Wu JW, Herman M, Liu L (2013). Small misfolded tau species are internalized via bulk endocytosis and anterogradely and retrogradely transported in neurons. J Biol Chem.

[27] Gómez-Ramos A, Díaz-Hernández M, Rubio A (2008). Extracellular tau promotes intracellular calcium increase through M1 and M3 muscarinic receptors in neuronal cells. Mol Cell Neurosci.

[28] Holmes BB, DeVos SL, Kfoury N (2013). Heparan sulfate proteoglycans mediate internalization and propagation of specific proteopathic seeds. Proc Natl Acad Sci USA.

[29] Calafate S, Flavin W, Verstreken P, Moechars D (2016). Loss of Bin1 promotes the propagation of tau pathology. Cell Rep.

[30] Martini-Stoica H, Cole AL, Swartzlander DB (2018). TFEB enhances astroglial uptake of extracellular tau species and reduces tau spreading. J Exp Med.

[31] Perea JR, López E, Díez-Ballesteros JC (2019). Extracellular monomeric tau is internalized by astrocytes. Front Neurosci.

[32] Luo W, Liu W, Hu X (2015). Microglial internalization and degradation of pathological tau is enhanced by an anti-tau monoclonal antibody. Sci Rep.

[33] Bolós M, Llorens-Martín M, Jurado-Arjona J (2015). Direct evidence of internalization of tau by microglia in vitro and in vivo. J Alzheimers Dis.

[34] Alliot F, Godin I, Pessac B (1999). Microglia derive from progenitors, originating from the yolk sac, and which proliferate in the brain. Brain Res Dev Brain Res.

[35] Ginhoux F, Greter M, Leboeuf M (2010). Fate mapping analysis reveals that adult microglia derive from primitive macrophages. Science.

[36] Lawson LJ, Perry VH, Dri P, Gordon S (1990). Heterogeneity in the distribution and morphology of microglia in the normal adult mouse brain. Neuroscience.

[37] Alzheimer A (1910) Beiträge zur Kenntnis der pathologischen Neuroglia und ihrer Beziehungen zu den Abbauvorgängen im Nervengewebe. In: Histologische und histopathologische Arbeiten über die Grosshirnrinde mit besonderer Berücksichtigung der pathologischen Anatomie der Geisteskrankheiten. Gustav Fischer, Jena, pp 401–562

[38] Cras P, Kawai M, Siedlak S, Perry G (1991). Microglia are associated with the extracellular neurofibrillary tangles of Alzheimer disease. Brain Res.

[39] El Khoury J, Hickman SE, Thomas CA (1996). Scavenger receptor-mediated adhesion of microglia to beta-amyloid fibrils. Nature.

[40] Ising C, Venegas C, Zhang S (2019). NLRP3 inflammasome activation drives tau pathology. Nature.

[41] Perea JR, Ávila J, Bolós M (2018). Dephosphorylated rather than hyperphosphorylated tau triggers a pro-inflammatory profile in microglia through the p38 MAPK pathway. Exp Neurol.

[42] Kyriakis JM, Avruch J (2012). Mammalian MAPK signal transduction pathways activated by stress and inflammation: a 10-year update. Physiol Rev.

[43] Canovas B, Nebreda AR (2021). Diversity and versatility of p38 kinase signalling in health and disease. Nat Rev Mol Cell Biol.

[44] Bachstetter AD, Van Eldik LJ (2010). The p38 MAP kinase family as regulators of proinflammatory cytokine production in degenerative diseases of the CNS. Aging Dis.

[45] Hensley K, Floyd RA, Zheng NY (1999). p38 kinase is activated in the Alzheimer’s disease brain. J Neurochem.

[46] Goedert M, Hasegawa M, Jakes R (1997). Phosphorylation of microtubule-associated protein tau by stress-activated protein kinases. FEBS Lett.

[47] Reynolds CH, Nebreda AR, Gibb GM (1997). Reactivating kinase/p38 phosphorylates tau protein in vitro. J Neurochem.

[48] Reynolds CH, Betts JC, Blackstock WP (2000). Phosphorylation sites on tau identified by nanoelectrospray mass spectrometry: differences in vitro between the mitogen-activated protein kinases ERK2, c-Jun N-terminal kinase and P38, and glycogen synthase kinase-3beta. J Neurochem.

[49] Feijoo C, Campbell DG, Jakes R (2005). Evidence that phosphorylation of the microtubule-associated protein Tau by SAPK4/p38delta at Thr50 promotes microtubule assembly. J Cell Sci.

[50] Bolós M, Pallas-Bazarra N, Terreros-Roncal J (2017). Soluble tau has devastating effects on the structural plasticity of hippocampal granule neurons. Transl Psychiatry.

[51] Bolós M, Llorens-Martín M, Perea J (2017). Absence of CX3CR1 impairs the internalization of tau by microglia. Mol Neurodegener.

[52] Gómez-Ramos A, Díaz-Hernández M, Cuadros R (2006). Extracellular tau is toxic to neuronal cells. FEBS Lett.

[53] Gabandé-Rodríguez E, Keane L, Capasso M (2020). Microglial phagocytosis in aging and Alzheimer’s disease. J Neurosci Res.

[54] Brunello CA, Merezhko M, Uronen R-L, Huttunen HJ (2019). Mechanisms of secretion and spreading of pathological tau protein. Cell Mol Life Sci.

[55] Elliott JA, Winn WC (1986). Treatment of alveolar macrophages with cytochalasin D inhibits uptake and subsequent growth of Legionella pneumophila. Infect Immun.

[56] Zhang Y, Chen K, Sloan SA (2014). An RNA-sequencing transcriptome and splicing database of glia, neurons, and vascular cells of the cerebral cortex. J Neurosci.

[57] Zhang Y, Sloan SA, Clarke LE (2016). Purification and characterization of progenitor and mature human astrocytes reveals transcriptional and functional differences with mouse. Neuron.

[58] Yamada K, Cirrito JR, Stewart FR (2011). In vivo microdialysis reveals age-dependent decrease of brain interstitial fluid tau levels in P301S human tau transgenic mice. J Neurosci.

[59] Vaughan DW, Peters A (1974). Neuroglial cells in the cerebral cortex of rats from young adulthood to old age: an electron microscope study. J Neurocytol.

[60] Stancu I-C, Cremers N, Vanrusselt H (2019). Aggregated tau activates NLRP3-ASC inflammasome exacerbating exogenously seeded and non-exogenously seeded tau pathology in vivo. Acta Neuropathol.

[61] Davalos D, Grutzendler J, Yang G (2005). ATP mediates rapid microglial response to local brain injury in vivo. Nat Neurosci.

[62] Nimmerjahn A, Kirchhoff F, Helmchen F (2005). Resting microglial cells are highly dynamic surveillants of brain parenchyma in vivo. Science.

[63] Guay J, Lambert H, Gingras-Breton G (1997). Regulation of actin filament dynamics by p38 map kinase-mediated phosphorylation of heat shock protein 27. J Cell Sci.

[64] Jadhav S, Avila J, Schöll M (2019). A walk through tau therapeutic strategies. Acta Neuropathol Commun.

[65] Kelleher I, Garwood C, Hanger DP (2007). Kinase activities increase during the development of tauopathy in htau mice. J Neurochem.

[66] Bhaskar K, Konerth M, Kokiko-Cochran ON (2010). Regulation of tau pathology by the microglial fractalkine receptor. Neuron.

[67] Lee S, Xu G, Jay TR (2014). Opposing effects of membrane-anchored CX3CL1 on amyloid and tau pathologies via the p38 MAPK pathway. J Neurosci.

[68] Xia Z, Dickens M, Raingeaud J (1995). Opposing effects of ERK and JNK-p38 MAP kinases on apoptosis. Science.

[69] Mosher KI, Wyss-Coray T (2014). Microglial dysfunction in brain aging and Alzheimer’s disease. Biochem Pharmacol.

[70] Pluvinage JV, Haney MS, Smith BAH (2019). CD22 blockade restores homeostatic microglial phagocytosis in ageing brains. Nature.

[71] Reed-Geaghan EG, Savage JC, Hise AG, Landreth GE (2009). CD14 and Toll-like receptors 2 and 4 are required for fibrillar A-stimulated microglial activation. J Neurosci.

[72] Gerrits E, Brouwer N, Kooistra SM (2021). Distinct amyloid-β and tau-associated microglia profiles in Alzheimer’s disease. Acta Neuropathol.

[73] Andersson CR, Falsig J, Stavenhagen JB (2019). Antibody-mediated clearance of tau in primary mouse microglial cultures requires Fcγ-receptor binding and functional lysosomes. Sci Rep.

[74] Cavalli V, Vilbois F, Corti M (2001). The stress-induced MAP kinase p38 regulates endocytic trafficking via the GDI:Rab5 complex. Mol Cell.

[75] Fratti RA, Chua J, Deretic V (2003). Induction of p38 mitogen-activated protein kinase reduces early endosome autoantigen 1 (EEA1) recruitment to phagosomal membranes. J Biol Chem.

[76] Pelkmans L, Fava E, Grabner H (2005). Genome-wide analysis of human kinases in clathrin- and caveolae/raft-mediated endocytosis. Nature.

[77] Yang C, Zhu Z, Tong BCK (2020). A stress response p38 MAP kinase inhibitor SB202190 promoted TFEB/TFE3-dependent autophagy and lysosomal biogenesis independent of p38. Redox Biol.

[78] Jiang S, Bhaskar K (2020). Degradation and transmission of tau by autophagic-endolysosomal networks and potential therapeutic targets for tauopathy. Front Mol Neurosci.

[79] Perlmutter LS, Barron E, Chui HC (1990). Morphologic association between microglia and senile plaque amyloid in Alzheimer’s disease. Neurosci Lett.

[80] Du YS, Chen X, Fu J (1996). RAGE and amyloid-beta peptide neurotoxicity in Alzheimer’s disease. Nature.

[81] Bolmont T, Haiss F, Eicke D (2008). Dynamics of the microglial/amyloid interaction indicate a role in plaque maintenance. J Neurosci.

[82] Meyer-Luehmann M, Spires-Jones TL, Prada C (2008). Rapid appearance and local toxicity of amyloid-beta plaques in a mouse model of Alzheimer’s disease. Nature.

[83] Behrendt A, Bichmann M, Ercan-Herbst E (2019). Asparagine endopeptidase cleaves tau at N167 after uptake into microglia. Neurobiol Dis.

[84] Damani MR, Zhao L, Fontainhas AM (2011). Age-related alterations in the dynamic behavior of microglia. Aging Cell.

[85] Fang Y, Wang J, Yao L (2018). The adhesion and migration of microglia to β-amyloid (Aβ) is decreased with aging and inhibited by Nogo/NgR pathway. J Neuroinflammation.

[86] Benndorf R, Hayeß K, Ryazantsev S (1994). Phosphorylation and supramolecular organization of murine small heat shock protein HSP25 abolish its actin polymerization-inhibiting activity. J Biol Chem.

[87] Arrasate M, Pérez M, Avila J (2000). Tau dephosphorylation at tau-1 site correlates with its association to cell membrane. Neurochem Res.

[88] Rauch JN, Luna G, Guzman E (2020). LRP1 is a master regulator of tau uptake and spread. Nature.

[89] Wang P, Ye Y (2021). Filamentous recombinant human tau activates primary astrocytes via an integrin receptor complex. Nat Commun.

[90] Zhuang Z-Y, Kawasaki Y, Tan P-H (2007). Role of the CX3CR1/p38 MAPK pathway in spinal microglia for the development of neuropathic pain following nerve injury-induced cleavage of fractalkine. Brain Behav Immun.

[91] Doens D, Fernández PL (2014). Microglia receptors and their implications in the response to amyloid β for Alzheimer’s disease pathogenesis. J Neuroinflammation.

[92] Momtazmanesh S, Perry G, Rezaei N (2020). Toll-like receptors in Alzheimer’s disease. J Neuroimmunol.

[93] Gunawardana CG, Mehrabian M, Wang X (2015). The human tau interactome: binding to the ribonucleoproteome, and impaired binding of the proline-to-leucine mutant at position 301 (P301L) to chaperones and the proteasome. Mol Cell Proteomics.

[94] Stefanoska K, Volkerling A, Bertz J (2018). An N-terminal motif unique to primate tau enables differential protein-protein interactions. J Biol Chem.

[95] Luck K, Kim D-K, Lambourne L (2020). A reference map of the human binary protein interactome. Nature.

[96] Tomida T, Takekawa M, Saito H (2015). Oscillation of p38 activity controls efficient pro-inflammatory gene expression. Nat Commun.

[97] Kumar GS, Clarkson MW, Kunze MBA (2018). Dynamic activation and regulation of the mitogen-activated protein kinase p38. Proc Natl Acad Sci USA.

[98] Munoz L, Ralay Ranaivo H, Roy SM (2007). A novel p38 alpha MAPK inhibitor suppresses brain proinflammatory cytokine up-regulation and attenuates synaptic dysfunction and behavioral deficits in an Alzheimer’s disease mouse model. J Neuroinflammation.

[99] Roy SM, Grum-Tokars VL, Schavocky JP (2015). Targeting human central nervous system protein kinases: An isoform selective p38αMAPK inhibitor that attenuates disease progression in Alzheimer’s disease mouse models. ACS Chem Neurosci.

[100] Gee MS, Son SH, Jeon SH (2020). A selective p38α/β MAPK inhibitor alleviates neuropathology and cognitive impairment, and modulates microglia function in 5XFAD mouse. Alzheimers Res Ther.

[101] Maphis N, Jiang S, Xu G (2016). Selective suppression of the α isoform of p38 MAPK rescues late-stage tau pathology. Alzheimers Res Ther.

[102] Prins ND, Harrison JE, Chu H-M (2021). A phase 2 double-blind placebo-controlled 24-week treatment clinical study of the p38 alpha kinase inhibitor neflamapimod in mild Alzheimer’s disease. Alzheimers Res Ther.

