# Supplementary material for: p38 Inhibition Decreases Tau Toxicity in Microglia and Improves Their Phagocytic Function
Source: Mol Neurobiol. 2022 Jan 10;59(3):1632–48. doi: 10.1007/s12035-021-02715-0 (PMC8882095; doi:10.1007/s12035-021-02715-0)
Supplement: Supplementary file 1 — Supplementary file1 (PDF 39777 KB) [file 12035_2021_2715_MOESM1_ESM.pdf]

Supplementary information for:

p38 inhibition decreases tau toxicity in microglia and improves their phagocytic function

Perea JR[1,2,3], Bolós M[1,2], Cuadros R[1], García E[1], García-Escudero V[1,4], Hernández F[1,2,5], McManus RM[3], Heneka MT[3], Avila J[1,2,*]

[1]Department of Molecular Neuropathology, Centro de Biología Molecular "Severo Ochoa" (UAM-CSIC), Madrid 28049, Spain
[2]Center for Networked Biomedical Research on Neurodegenerative Diseases (CIBERNED), Madrid 28031, Spain
[3]German Center for Neurodegenerative Diseases (DZNE), Bonn 53127, Germany
[4]Department of Anatomy, Histology and Neurosciences, Faculty of Medicine, Universidad Autónoma de Madrid (UAM), Madrid 28029, Spain
[5]Department of Molecular Biology, Faculty of Sciences, Universidad Autónoma de Madrid (UAM), Madrid 28049, Spain

[*]Correspondence should be addressed to Jesús Avila. Centro de Biología Molecular "Severo Ochoa", Universidad Autónoma de Madrid (Campus de Cantoblanco), 1 Nicolás Cabrera st, 28049, Madrid (Spain). Phone number: +34-911964564. javila@cbm.csic.es

Supplementary figures

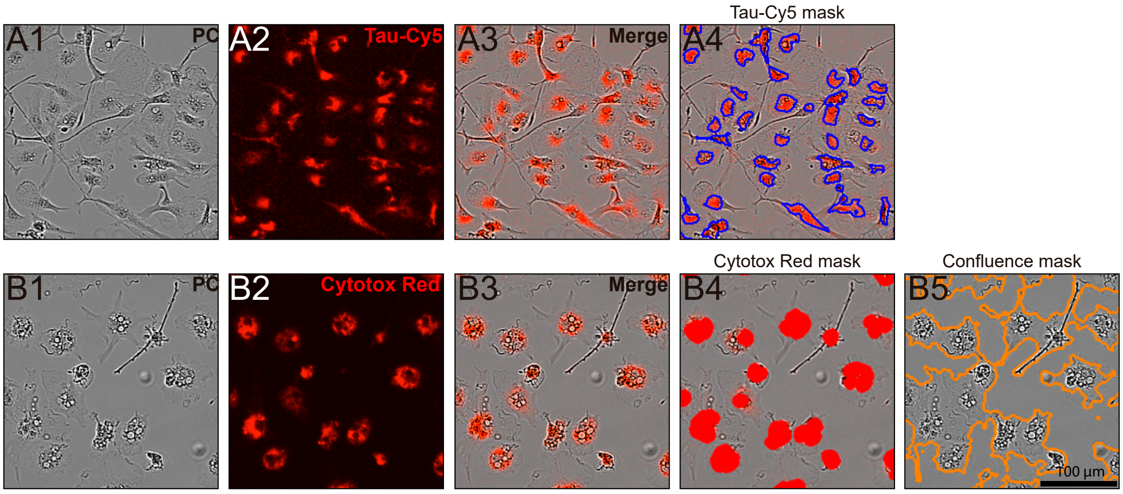

Supplementary figure S1 Depiction of the masks that were used for automated analysis of Tau-Cy5 internalization (A4), cytotoxicity (B4), and confluence (B5). Scale bar: 100 µm. PC: phase contrast.

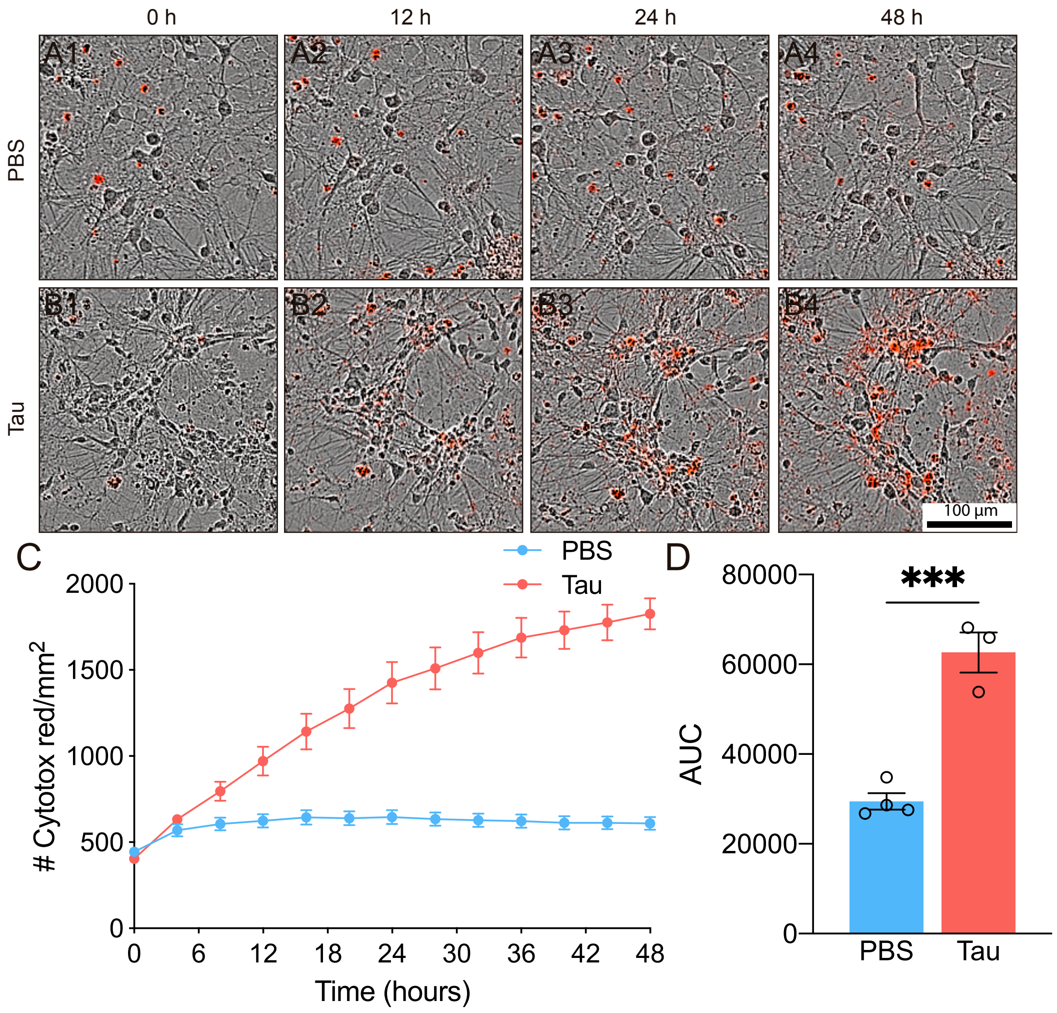

Supplementary figure S2 Tau is toxic to neuronal cells. Representative images of neurons treated with PBS (control) (A) or tau protein (B) for 48 h. Cytotox Red labeling indicates cytotoxicity. (C-D) Cytotoxicity analysis reveals that tau protein is toxic for neurons. *n* = 4. Graphs show mean ± SEM. ***$p<0.001$ from one-way ANOVA. Scale bar: 100 µm. AUC: area under the curve, PBS: phosphate-buffered saline.

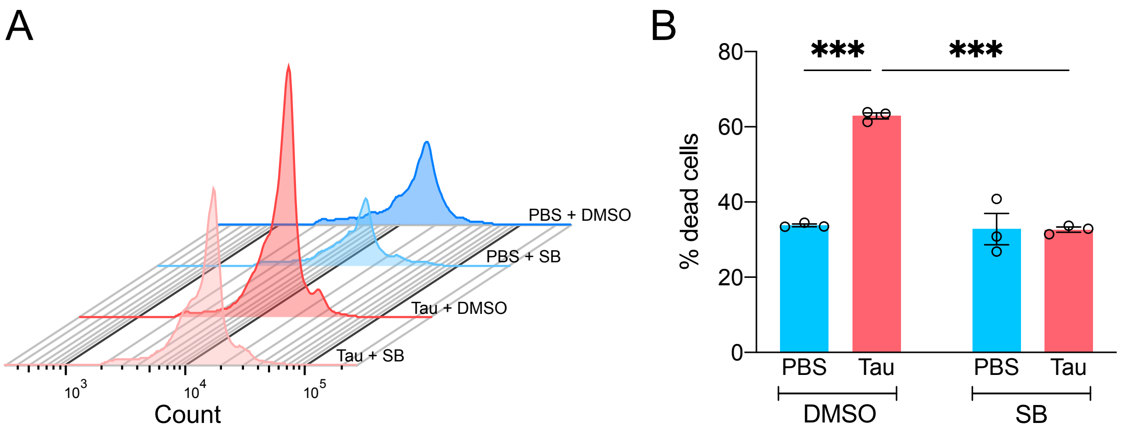

Supplementary figure S3 p38 inhibition decreases tau-mediated cytotoxicity in microglia. Flow cytometry histogram (A) and quantification of the number of dead cells (B) in microglia treated for 6 h with PBS (control) or tau in the absence or presence of SB203580 (SB). $n = 3$. Graph shows mean ± SEM. ***$p<0.001$ from two-way ANOVA. DMSO: dimethyl sulfoxide, PBS: phosphate-buffered saline.

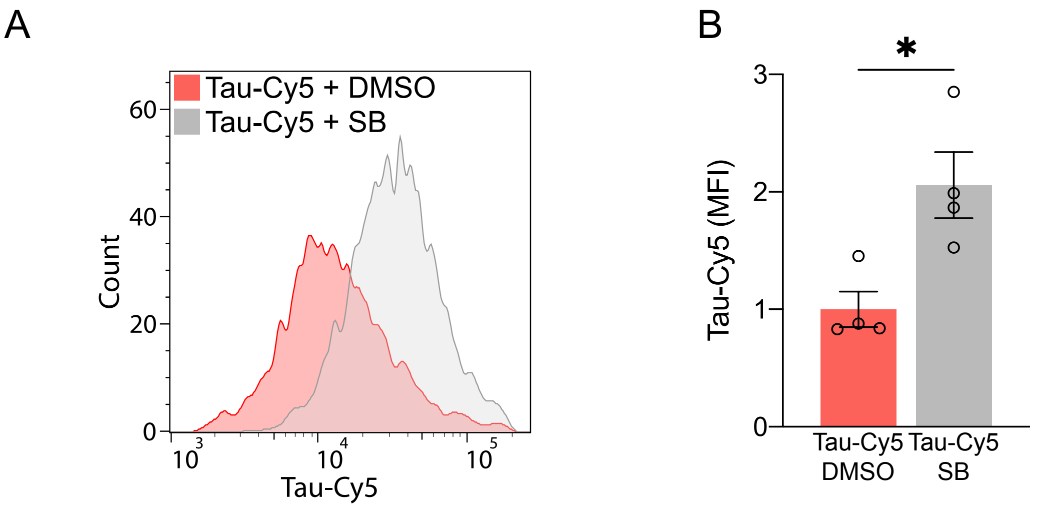

Supplementary figure S4 p38 inhibition enhances tau phagocytosis by microglia. Flow cytometry histogram (A) and quantification of relative mean fluorescence intensity (MFI) of tau-Cy5 (B) in microglia treated for 6 h with DMSO (control) or SB203580 (SB). $n = 4$. Graph shows mean ± SEM. *$p<0.05$ from Student's *t*-test (two-tailed). DMSO: Dimethyl sulfoxide, MFI: mean fluorescence intensity.

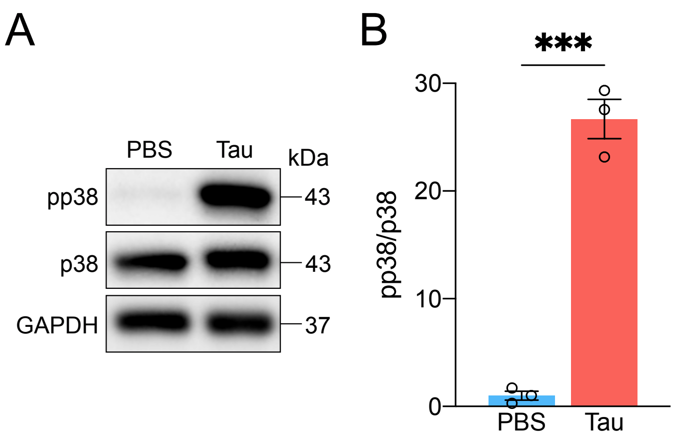

Supplementary figure S5 Tau activates p38 MAPK in *Cx3cr1*$^{-/-}$ microglia. Western blot (A) and quantification of p38 activity (B) in microglia treated for 30 min with PBS (control) and tau. $n = 3$. Graphs show mean ± SEM. ***p<0.001 from Student's t-test (two-tailed). GAPDH: Glyceraldehyde-3-phosphate dehydrogenase, PBS: phosphate-buffered saline.

Supplementary tables

Antibody	Host	Manufacturer (ref.)	RRID	Dilution
**Anti-pp38**	Rabbit (p)	Cell Signaling (9211)	RRID:AB_331641	1:1,000
**Anti-p38**	Rabbit (p)	Cell Signaling (9212)	RRID:AB_330713	1:1,000
**Anti-pMK2**	Mouse (m)	Santa Cruz (sc-293140)	Not available	1:1,000
**Anti-MK2**	Mouse (m)	Santa Cruz (sc-393609)	Not available	1:100
**Anti-pHsp27**	Rabbit (m)	Cell Signaling (9709)	RRID:AB_11217429	1:250
**Anti-Hsp27**	Rabbit (p)	Cell Signaling (2442)	RRID:AB_2233273	1:250
**Anti-GFP**	Rabbit (p)	Thermo Fisher (A-11122)	RRID:AB_221569	1:1,000
**Anti-Tau12**	Mouse (m)	Merck (MAB2241)	RRID:AB_1977340	1:300
**Anti-GAPDH**	Mouse (m)	Abcam (ab8245)	RRID:AB_2107448	1:1,000
**Anti-actinβ**	Mouse (m)	Sigma Aldrich (A5441)	RRID:AB_476744	1:1,000
**Anti-mouse HRP**	Goat (p)	Dako (P0447)	RRID:AB_2617137	1:2,000
**Anti-rabbit HRP**	Goat (p)	Dako (P0448)	RRID:AB_2617138	1:2,000

Supplementary Table S1 Primary and secondary antibodies. GAPDH: Glyceraldehyde-3-phosphate dehydrogenase, GFP: Green fluorescent protein, HRP: Horseradish peroxidase, Hsp27: Heat shock protein 27, MK2: MAPK-activated protein kinase 2, m: monoclonal, p: polyclonal, RRID: research resource identifier.

**WT (forward)**	GTCTTCACGTTCGGTCTGGT
**Common (reverse)**	CCCAGACACTCGTTGTCCTT
**Mutant (forward)**	CTCCCCCTGAACCTGAAAC

Supplementary Table S2 PCR primers for genotyping.

Supplementary Video S1 Tau exerts a toxic effect on microglia. Representative time-lapse video of primary microglia cultures treated with PBS (left), LPS (middle) and tau (right) for 48 h. Orange lines delimit the contour of the cells, while red dots indicate cytotoxicity.

Supplementary Video S2 Cytochalasin D prevents microglia-mediated tau internalization. Representative time-lapse video of primary microglia cultures treated with tau-Cy5 in the absence (left) or presence (right) of Cytochalasin D for 24 h. Blue lines show tau-Cy5 internalization.

Supplementary Video S3 Phagocytosis inhibition decreased tau-mediated cytotoxicity in microglia. Representative time-lapse video of primary microglia cultures treated with PBS + DMSO (upper left), Tau + DMSO (upper right), PBS + CytoD (lower left) and Tau + CytoD (lower right) for 24 h. Orange lines delimit the contour of the cells, while red dots indicate cytotoxicity.

Suplemmentary Video S4 p38 inhibition decreased tau-mediated cytotoxicity in microglia. Representative time-lapse video of primary microglia cultures treated with PBS + DMSO (upper left), Tau + DMSO (upper right), PBS + SB (lower left) and Tau + SB (lower right) for 24 h. Orange lines delimit the contour of the cells, while red dots indicate cytotoxicity.

Supplementary Methods

Primary cultures of neurons

Neurons were cultured from C57BL/6J (Charles River. RRID:MGI:6151402) mouse embryos (E16). Brains were dissected in cold Ca^{2+}/Mg^{2+}-free HBSS and stripped of meninges. The tissue was then digested with 0.025% trypsin and 1 mg/ml DNase (Roche. Cat#11284932001) for 5 min at 37°C. After carefully removing as much volume as possible, trypsinization was stopped by the addition of Neurobasal (Gibco. Cat#12348-017) supplemented with 1% B27 (Gibco. Cat#17502-048), 10% FBS (Gibco. Cat#26140-079), 1% Glutamax (Gibco. Cat#35050-038), 100 U/mL of penicillin and 0.1 mg/mL of streptomycin (Gibco. Cat#15240-062). A single-cell suspension was obtained by repeated pipetting. Subsequently, cells were passed through a 40 µm nylon filter and seeded (100,000 cells/well) into M24 plates coated with 10 µg/ml poly-L-lysine (Sigma Aldrich. Cat#P9155), which were maintained at 37°C in humidified 5% CO_2–95% air. The next day, the plating medium was discarded and fresh serum-free medium was added. The culture medium was partially replaced every two days until *in vitro* day 8 for experimentation. Neurons were then treated with 0.5 µM tau or PBS (control). The plate was immediately introduced into the Incucyte S3 system (Sartorius) and 9 images/well were taken every 6 h for 48 h with a 10x objective. Image processing and quantification were carried out by the integrated controller unit (Sartorius) using the Incucyte S3

v.2017A software. Cell death events were visualized by adding 250 nM Cytotox Red reagent (Sartorius. Cat#4632), following the manufacturer's instructions.

Tau internalization assay (FACS)

300,000 cells/well were seeded on an M24 plate. After 2 days, cells were pre-treated for 30 min with 20 µM SB203580 (Sigma Aldrich. Cat#S8307) or vehicle (DMSO), and 0.5 µM tau-Cy5 or PBS-Cy5 (control) was subsequently added for 6 h. Cells were then washed three times with PBS to remove excess tau. Trypsin was then used to lift cells from the plate and cells were again washed twice with PBS by centrifugation (5 min, 150 *g*, 4°C). Finally, cells were resuspended in 200 µl of PBS and analyzed using a FACSCanto II flow cytometer (BD) and FlowJo v.10.4 (RRID:SCR_008520). Dead cells were identified using 1 µg/ml of DAPI (Merck. Cat#268298) in order to exclude them from the analysis.

Quantification of dying cells

300,000 cells/well were seeded on an M24 plate. After 2 days, cells were pre-treated for 30 min with 20 µM SB203580 (Sigma Aldrich. Cat#S8307) or vehicle (DMSO), and 0.5 µM tau or PBS (control) was subsequently added for 6 h. Cells were then washed three times with PBS to remove excess tau. Trypsin was then used to lift cells from the plate and cells were again washed twice with PBS by centrifugation (5 min, 150 *g*, 4°C). Finally, cells were resuspended in 200 µl of PBS and stained with 1 µg/ml of DAPI (Merck. Cat#268298) prior to analysis using a FACSCanto II flow cytometer (BD) and FlowJo v.10.4 (RRID:SCR_008520).

Cx3cr1$^{-/-}$ animals and genotyping

The mouse lacking CX3CR1 (B6.129P2(Cg)-*Cx3cr1^{tm1Litt}*/J) was generated by Jung *et al.* and donated to The Jackson Laboratory (RRID:IMSR_JAX:005582). *Cx3cr1* gene expression was interrupted by insertion of the EGFP coding sequence, thus replacing the first 390 bp of exon 2. This deleted region codes for the N-terminal fragment of CX3CR1, which is essential for the interaction of this receptor with its ligand (CX3CL1). qRT-PCR and flow cytometry analysis confirmed the absence of endogenous protein expression and the presence of GFP in monocytes, dendritic cells, NK cells and microglia (Jung *et al.* 2000).

The line was kept in homozygosis and the genotype was determined by nested PCR from genomic DNA obtained from a small portion of the tail. To this end, the tissue was digested with 150 µl of 50 mM NaOH in a thermomixer (1 h, 450 rpm, 99°C). The digestion was neutralized with 15 µl of 1 M Tris-HCl pH 8 and subsequently centrifuged (10 min, 18,400 *g*, room temperature). The components of the GoTaq G2 Flexi DNA Polymerase kit (Promega. Cat#M7801), the dNTP mix (Thermo Fisher. Cat#10297-018)

and the primers (Supplementary Table S2 online) were added as follows: 2 µl of genomic DNA; 2.4 µl of 5× green buffer; 0.96 µl of 25 mM $MgCl_2$; 0.48 µl of 5 mM dNTP mix; 0.3 µl of each of the 20 µM primers; 0.1 µl Taq polymerase (5U/µl); and Milli-Q water up to 10 µl volume. Nested PCR had 2 rounds of amplification after an initial time of 2 min at 94°C. The first round of amplification consisted of 11 cycles of denaturation (94°C, 20 s), annealing (65°C(-0.5°C/cycle), 15 s) and extension (68°C, 10 s). The second round consisted of 29 cycles of denaturation (94°C, 15 s), annealing (60°C, 15 s) and extension (72°C, 10 s). Finally, the elongation time was for 2 min at 72°C. The PCR product resulted in a 500 bp band (*Cx3cr1*$^{-/-}$) and a 410 bp band (WT).

References

Jung S., Aliberti J., Graemmel P., Sunshine M. J., Kreutzberg G. W., Sher A., Littman D. R. (2000) Analysis of fractalkine receptor CX3CR1 function by targeted deletion and green fluorescent protein reporter gene insertion. *Mol. Cell. Biol.* **20**, 4106–14.
